# Decoding Salinity Tolerance in *Salicornia europaea* L.: Image-Based Oxidative Phenotyping and Histochemical Mapping of Pectin and Lignin

**DOI:** 10.3390/plants14193055

**Published:** 2025-10-02

**Authors:** Susana Dianey Gallegos Cerda, Aleksandra Orzoł, José Jorge Chanona Pérez, Josué David Hernández Varela, Agnieszka Piernik, Stefany Cárdenas Pérez

**Affiliations:** 1 Department of Geobotany and Landscape Planning, Faculty of Biological and Veterinary Sciences, Nicolaus Copernicus University in Toruń, Lwowska 1, 87-100 Toruń, Poland; 2 Departamento de Ingeniería Bioquímica, Escuela Nacional de Ciencias Biológicas, Instituto Politécnico Nacional, Av. Wilfrido Massieu, Gustavo A. Madero, Ciudad de México 07738, Mexico

**Keywords:** halophyte, lipid peroxidation—Schiff’s, hydrogen peroxide—DAB, population salt-tolerance, cell polymers, computer vision system

## Abstract

Halophytes such as *Salicornia europaea* rely on biochemical and structural mechanisms to survive in saline environments. This study aimed to evaluate oxidative stress and structural defense responses in four inland populations—Poland (Inowrocław, Ciechocinek), Germany (Salzgraben-Salzdahlum, Salz), and Soltauquelle (Soltq)—subjected to 0, 200, 400, and 1000 mM NaCl, using non-destructive, image-based approaches. Lipid peroxidation was assessed via malondialdehyde (MDA) detected with Schiff’s reagent, and hydrogen peroxide (H_2_O_2_) accumulation was visualized with 3,3′-diaminobenzidine (DAB). Roots and shoots were analyzed through colour image analysis and quantified using a computer vision system (CVS). MDA accumulation revealed population-specific differences, with Salz tending to exhibit lower peroxidation, characterized by lower L* ≈ 42–43 and higher b* ≈ 37–18 in shoots at 200–400 mM, which may reflect a potentially more effective salt-management strategy. Although H_2_O_2_ responses deviated from a direct salinity-dependent trend, particularly in the tolerant Salz and Soltq populations, both approaches effectively tracked population-specific adaptation, with German populations displaying detectable basal H_2_O_2_ levels, consistent with its multifunctional signalling role in salt management and growth regulation. Structural defences were further explored through histochemical mapping and image analysis of pectin and lignin distribution, which revealed population-specific patterns consistent with cell wall remodelling under stress. Non-destructive, image-based methods proved effective for detecting oxidative and structural responses in halophytes. Such a non-destructive, cost-efficient, and reproducible approach can accelerate the identification of salt-tolerant ecotypes for saline agriculture and reinforce *S. europaea* as a model species for elucidating salt-tolerance mechanisms.

## 1. Introduction

Halophytes are well-known for their ability to adapt to high salinity environments through various phenotypic and metabolic adjustments [1]. One of their primary adaptive mechanisms involves the controlled accumulation and sequestration of ions, enabling osmotic regulation within tissues under saline conditions [2]. Understanding physiological and biochemical responses underlying salt tolerance is crucial to understanding the potential of halophytes as sustainable crops in marginal environments [3].

Salinity is a major abiotic factor that induces oxidative stress in halophytes. Reactive oxygen species (ROS) generated under high salt conditions act as essential signals at moderate levels, but cause cellular damage when excessive [4,5]. Lipid peroxidation, a key consequence, produces malondialdehyde (MDA), which can be quantified using Schiff’s reagent [6,7]. Hydrogen peroxide (H_2_O_2_), another central ROS with dual signalling and damaging roles, can be visualized by diaminobenzidine (DAB) staining [8,9,10]. *Salicornia europaea* L., an extreme halophyte of Northern Europe, shows high plasticity to salinity [11]. In a recent work, Orzoł et al. [12] examined MDA and antioxidant enzyme responses across populations under NaCl gradients (0–1000 mM), Duan et al. [13] revealed changes in antioxidant metabolites and lipids under saline conditions, and Cárdenas-Pérez et al. [14] reported peroxidase activity and pigment variations under salinity.

Lipid peroxidation serves as a key biomarker of cellular damage under stress conditions [15]. Traditional detection methods, such as the thiobarbituric acid assay, suffer from interference by compounds like anthocyanins, affecting accuracy [16,17]. More advanced techniques, including HPLC-GC and other spectroscopies such as UV spectroscopy, offer higher sensitivity but often lack specificity due to the complexity of biological matrices and diverse lipid compositions [6,7]. In contrast, Schiff’s reagent provides high specificity for aldehydes derived from lipid peroxides, enabling precise detection even at low concentrations with minimal interference.

In addition to oxidative markers, structural cell-wall polymers such as pectin and lignin play key roles in salinity tolerance [18,19,20]. Pectin contributes to ion binding and wall flexibility, facilitating controlled ion transport and stress signalling, while lignin enhances mechanical strength and limits cellular permeability, thereby protecting tissues from ion toxicity and oxidative damage. These well-established functions support the structural resilience of halophyte crops, where cell wall remodelling supports growth and survival in saline environments. Histochemical staining allows the localization of pectin and lignin within tissues, providing insight into the anatomical and biochemical strategies that plants, including halophytes, employ to cope with salinity [21,22].

In this context, we hypothesize that salinity-induced oxidative stress in four distinct inland populations of *S. europaea* results in tissue-specific patterns of lipid peroxidation and H_2_O_2_. These patterns can be visualized through image analysis of Schiff’s and DAB reagents staining, potentially revealing population-specific tolerance mechanisms.

Therefore, the objectives of this study were as follows: 1. Assess oxidative stress responses in shoot and root tissues of different inland populations of *S. europaea* under salt stress using non-destructive, image-based detection of aldehydes produced during lipid peroxidation. 2. Examine the spatial distribution of pectin and lignin under salinity through histochemical staining combined with image analysis to elucidate the role of cell-wall modifications in salt tolerance. 3. Evaluate the potential of image analysis as a cost-effective tool to identify population-specific responses, distinguish salt-tolerant from salt-sensitive phenotypes, and capture spatial variation in salt accumulation within plant tissues.

## 2. Results

### 2.1. Lipid Peroxidation Visualization

**Figure 1:**
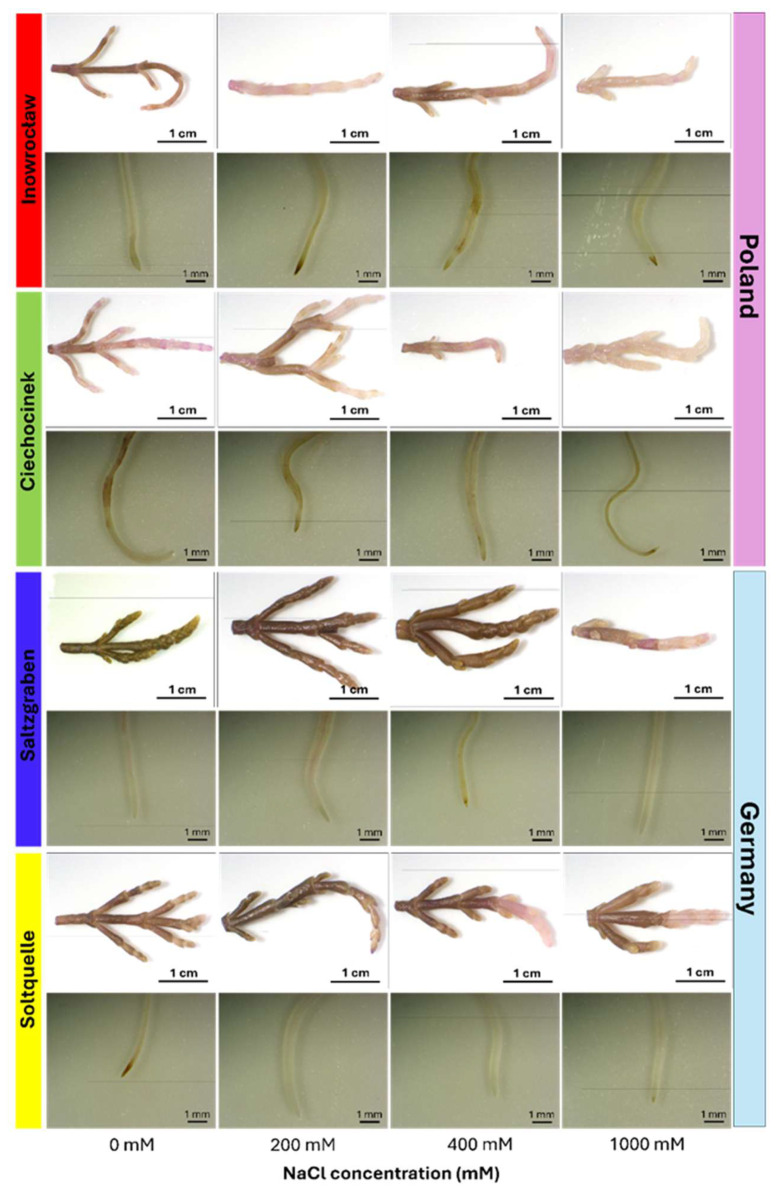
Representative images of shoots (**top row**) and roots (**bottom row**) of four *S. europaea* populations (Inowrocław, Ciechocinek, Salzgraben, and Soltauquelle) treated with different NaCl concentrations (0, 200, 400, and 1000 mM). Samples were stained with Schiff’s reagent to detect MDA-lipid peroxidation, where pale pink colouration indicates the presence of malondialdehyde (MDA) (n = 3).

Representative images of shoots and roots exposed to 0, 200, 400, and 1000 mM NaCl are shown in Figure 1. Schiff’s reagent staining revealed pink colouration from lipid peroxidation (MDA), with clear population contrasts. Polish populations (Inow, Ciech) showed strong pink staining in shoots from 200 mM onward, intensifying at higher salinity levels, while the German populations (Salz, Soltq) maintained darker shoots with noticeable changes only at 400–1000 mM. Root tissues were less responsive overall; even at 1000 mM, roots of all populations resembled controls, with only slight staining increases in Salz and Soltq (Figure 1; Table 1 and Table 2).

#### 2.1.1. Shoot Colorimetric Parameters Through CIELab, Schiff’s/MDA

**Figure 2:**
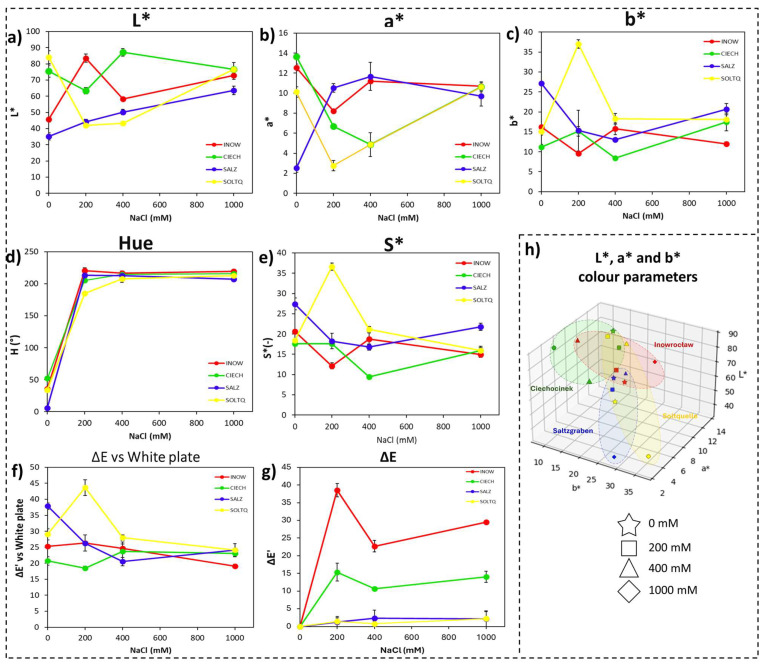
Colourimetric changes in shoots of four *S. europaea* populations (INOW, Inowrocław; CIECH, Ciechocinek; SALZ, Salzgraben; SOLTQ, Soltauquelle) subjected to salinity treatments (0, 200, 400, and 1000 mM NaCl). Lipid peroxidation was visualized with Schiff’s reagent, and colour parameters were quantified using computer vision analysis: (**a**) L* (lightness), (**b**) a* (green–red component), (**c**) b* (blue–yellow component), (**d**) hue angle, (**e**) chroma or saturation (S*), (**f**) colour difference (ΔE) relative to a white standard, and (**g**) colour difference (ΔE′) between successive salinity treatments. (**h**) Three-dimensional distribution of CIELab parameters (L*, a*, b*) shows population-specific shifts in colour attributes asociated with salt-induced lipid peroxidation. Ellipsoids highlight the clustering of responses for each population. Bars indicate means ± SD of replicates (n = 3).

Colorimetric parameters derived from CVS image analysis are presented in Figure 2 and Table 1, with detailed MANOVA statistics provided in Supplementary Table S1. For the Schiff’s reagent (MDA) assay, a two-way MANOVA on the basic CIELab variables (L*, a*, b*) revealed a significant multivariate effect of Population (Wilks’ Λ = 0.384, F = 4.96, *p* < 0.001), whereas Salinity (*p* = 0.278) and the Population × Salinity interaction (*p* = 0.266) were not significant, indicating that population differences dominated the colour variation associated with lipid peroxidation.

Consistent with the MANOVA results, two-way ANOVA for each CIELab parameter (Table 1; Supplementary Table S2) showed highly significant effects of population (*p* < 0.001) and a population × salinity interaction (*p* < 0.05), confirming that population identity was the main driver of colour variation. Polish populations (Inow, Ciech) displayed higher lightness and stronger overall colour shifts (Figure 2a,f,g), whereas the German populations (Salz, Soltq) maintained lower L* and more compact colour clusters. Among the derived metrics, Hue and Chroma followed similar population-specific trajectories, with Soltq showing a transient enhancement at 200 mM and Salz remaining comparatively stable (Figure 2d,e). The red–green (a*) and yellow–blue (b*) axes further reflected these trends (Figure 2b,c). Three-dimensional CIELab plots (Figure 2h) highlighted the contrast, revealing broader dispersion in the Polish populations and tighter clustering in the German populations, consistent with greater oxidative instability in Poland and higher colour stability in Germany.

#### 2.1.2. Root Colorimetric Parameters Through CIELab, Schiff’s/MDA

**Figure 3:**
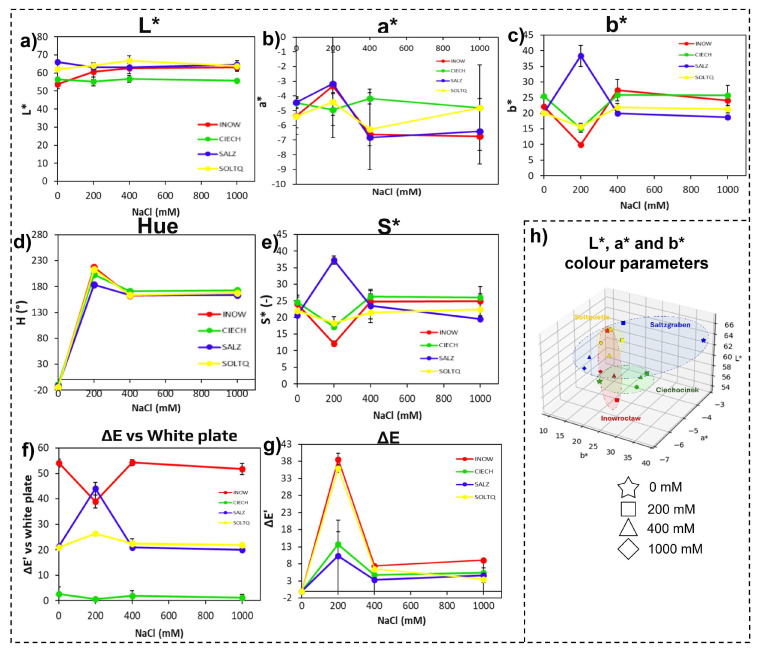
Colourimetric changes in root tissues of four *S. europaea* populations, Inowrocław (INOW), Ciechocinek (CIECH), Salzgraben (SALZ), and Soltauquelle (SOLTQ), exposed to salinity treatments (0, 200, 400, and 1000 mM NaCl). Lipid peroxidation was visualized using Schiff’s reagent, and colour parameters were extracted from images with a computer vision system (CVS): (**a**) lightness (L*), (**b**) red–green component (a*), (**c**) yellow–blue component (b*), (**d**) hue angle, (**e**) chroma (S*), (**f**) colour difference (ΔE) relative to a white standard, and (**g**) intra-treatment colour difference (ΔE′) between successive salinity levels. (**h**) Three-dimensional distribution of CIELab parameters (L*, a*, and b*) shows population-specific colour shifts associated with lipid peroxidation under NaCl stress. Ellipsoids highlight the clustering of responses for each population, illustrating differences in oxidative stress responses. Bars indicate means ± SD of replicates (n = 3).

Root colorimetric responses are shown in Figure 3 and summarized in Table 2. In Figure 3a, lightness (L*) values remained relatively stable across populations, with Soltq showing a slight increase at high salinity, while Inow and Ciech maintained lower and flatter responses. Figure 3b illustrates the red–green axis (a*), where Inow and Ciech shifted towards more negative values under salinity, reflecting stronger greenish tones, whereas Salz and Soltq showed more moderate declines. Figure 3c shows the yellow–blue axis (b*), which peaked at 200 mM in Salz and remained higher than other populations, while Ciech and Soltq showed comparatively muted changes.

Hue values (Figure 3d) increased sharply after 200 mM in all populations and then stabilized, but the rise was more pronounced in Inow and Soltq. Chroma (Figure 3e) declined gradually with increasing salinity in most cases, yet Salz peaked at 200 mM, and Inow and Ciech showed a steeper loss of saturation, contrasting with Salz and Soltq, which retained higher values at intermediate treatments.

The colour difference versus a white reference (ΔE, Figure 3f) revealed the strongest shifts in Inow and Soltq at 200 mM, while Salz and Ciech exhibited more moderate changes. The intra-treatment colour difference (ΔE’, Figure 3g) highlighted greater variation in Inow and Salz.

Finally, the 3D CIELab plots (Figure 3h) revealed broad dispersion of Inow values, suggesting greater instability, while Ciech formed a tighter cluster, indicating more consistent colour behaviour. Soltq displayed a widespread distribution across axes, while Salz remained the most compact and stable of all four populations.

### 2.2. Hydrogen Peroxide Accumulation

**Figure 4:**
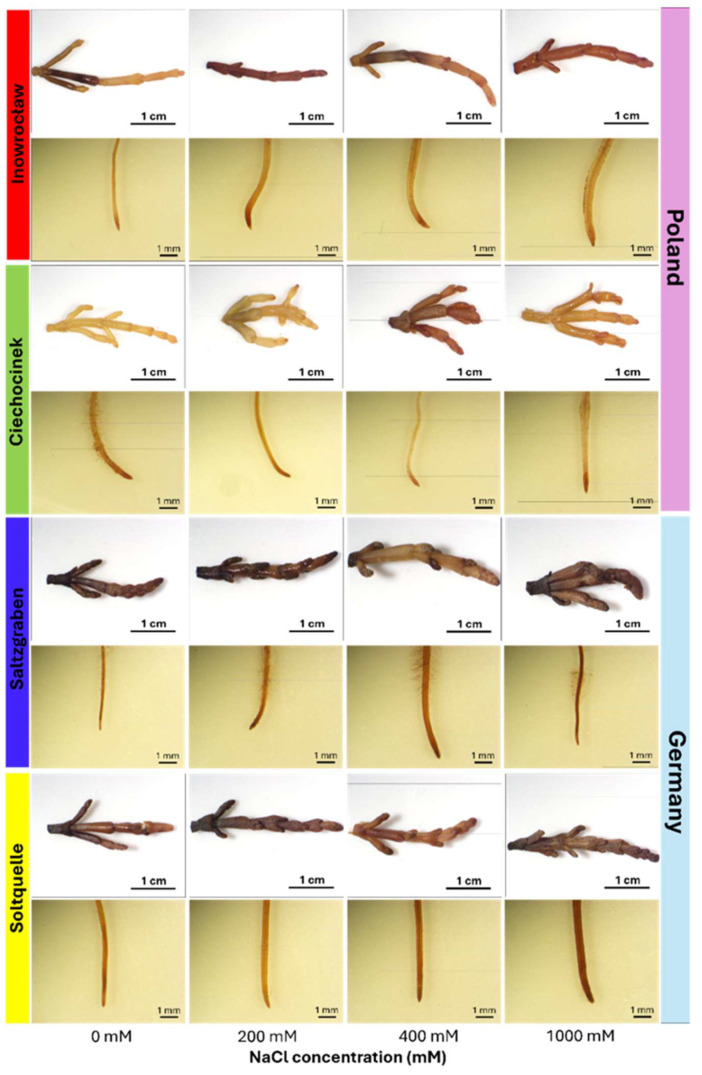
Localization of hydrogen peroxide (H_2_O_2_) using 3,3′-diaminobenzidine (DAB) reagent in shoots and roots of *S. europaea* from four populations—Inowrocław, Ciechocinek (Poland), Salzgraben, and Soltauquelle (Germany)—exposed to increasing concentrations of NaCl (0, 200, 400, and 1000 mM). H_2_O_2_ accumulation was visualized via 3,3′-diaminobenzidine (DAB) staining; the brown precipitate indicates the presence of H_2_O_2_. Each row corresponds to a population and tissue type ((**upper row**): shoot; (**lower row**): root) (n = 3).

Detection of H_2_O_2_ by DAB staining is shown in Figure 4, with detailed MANOVA statistics provided in Supplementary Table S1. A two-way MANOVA on the basic CIELab variables (L*, a*, b*) revealed significant multivariate effects of Population (Wilks’ Λ = 0.269, F = 7.37, *p* < 0.001), Salinity (Wilks’ Λ = 0.673, F = 6.15, *p* = 0.0016), and a strong Population × Salinity interaction (Wilks’ Λ = 0.409, F = 4.57, *p* = 0.0001), indicating that salinity-driven changes in H_2_O_2_ colour parameters were strongly population dependent. Consistent with these multivariate results, two-way ANOVA for individual CIELab parameters (Table 2) also showed significant effects of population and Population × Salinity interaction (*p* < 0.05). In shoots and roots of Inow and Ciech, brown colouration was faint under 0 mM and increased progressively with higher salinity, reaching the greatest intensity at 1000 mM. Both German populations (Salz and Soltq) exhibited clear basal H_2_O_2_ staining under 0 mM conditions and maintained stronger colouration than the Polish populations across all treatments. Soltq showed the highest intensity overall, particularly in the roots, whereas Salz displayed intermediate but consistently elevated colouration compared with Inow and Ciech.

#### 2.2.1. Shoot Colourimetric Parameters Through CIELab, DAB/H_2_O_2_

**Figure 5:**
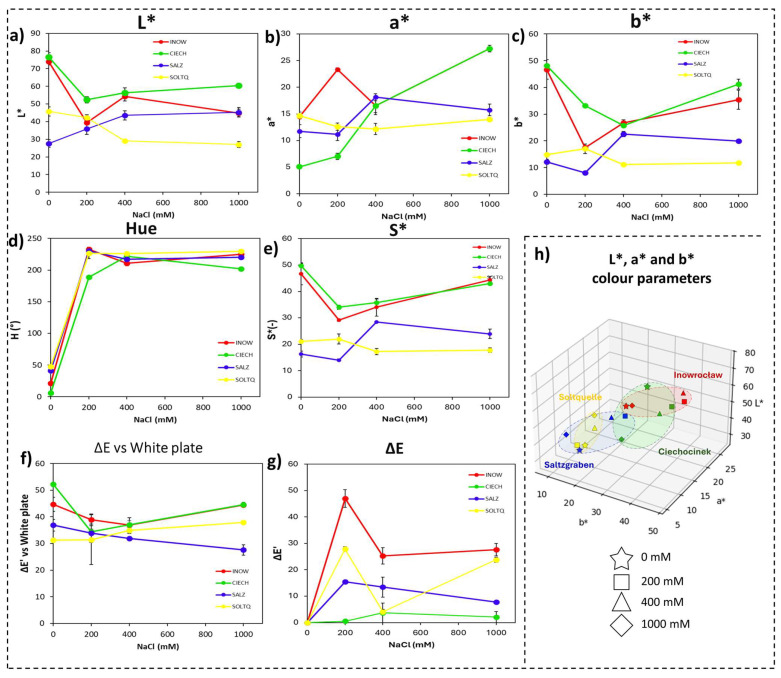
Colour changes in shoots of four *S. europaea* populations, INOW (Inowrocław), CIECH (Ciechocinek), SALZ (Salzgraben), and SOLTQ (Soltauquelle), subjected to four NaCl concentrations (0, 200, 400, and 1000 mM) for H_2_O_2_ detection using 3,3′-diaminobenzidine (DAB) reagent. Colour parameters were extracted from images using a Computer Vision System (CVS). Panels show: (**a**) lightness (L*), (**b**) red–green component (a*), (**c**) yellow–blue component (b*), (**d**) hue angle, (**e**) chroma (saturation; S*), (**f**) colour difference (ΔE) relative to a white standard, and (**g**) intra-treatment colour difference (ΔE′) between successive salinity treatments. (**h**) Three-dimensional distribution of CIELab parameters (L*, a*, b*) illustrates population-specific colour changes associated with H_2_O_2_ accumulation under NaCl treatments. Ellipsoids highlight the clustering of each population’s response, illustrating shifts in colour attributes related to salt-induced oxidative stress. Bars indicate means ± SD of replicates (n = 3).

Colour parameters associated with H_2_O_2_ detection are presented in Figure 5 and Table 2. Lightness (L*, Figure 5a), red–green (a*, Figure 5b), and yellow–blue (b*, Figure 5c) axes followed trends similar to those observed for Schiff’s staining, with clear colour changes even in small tissue regions. Overall, Polish populations displayed higher L*, a* and b* values than German ones, especially at higher salinities. Hue (Figure 5d) and Chroma (Figure 5e) showed distinct tendencies among populations. Colour differences relative to the white reference (ΔE, Figure 5f) and intra-treatment colour differences (ΔE′, Figure 5g) revealed marked population-specific shifts, especially in Inow and Ciech, which exhibited stronger intra-treatment changes at higher salinities. The 3D distribution of L*, a*, and b* values (Figure 5h) showed clearly separated clusters between Polish and German populations.

#### 2.2.2. Root Colourimetric Parameters Through CIELab, DAB/H_2_O_2_

**Figure 6:**
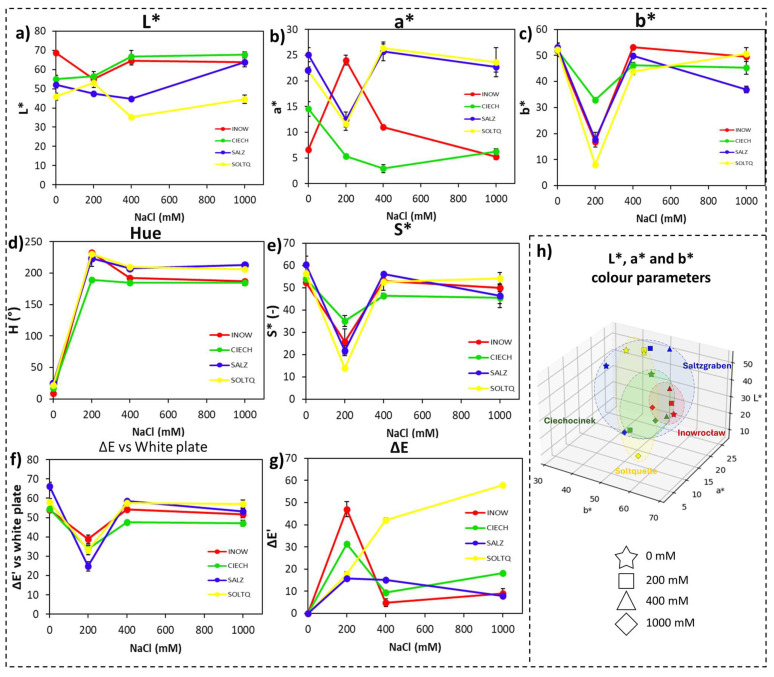
Colour changes in roots of four *S. europaea* populations—INOW (Inowrocław), CIECH (Ciechocinek), SALZ (Salzgraben), and SOLTQ (Soltauquelle)—subjected to four NaCl concentrations (0, 200, 400, and 1000 mM) for H_2_O_2_ detection using 3,3′-diaminobenzidine (DAB) reagent. Colour parameters were extracted from images using a Computer Vision System (CVS). Panels show: (**a**) lightness (L*), (**b**) red–green component (a*), (**c**) yellow–blue component (b*), (**d**) hue angle, (**e**) chroma (saturation; S*), (**f**) colour difference (ΔE) relative to a white standard, and (**g**) intra-treatment colour difference (ΔE′) between successive salinity treatments. (**h**) Three-dimensional distribution of CIELab parameters (L*, a*, b*) illustrates population-specific colour changes associated with H_2_O_2_ accumulation under NaCl treatments. Ellipsoids highlight the clustering of each population’s response, showing shifts in colour attributes related to salt-induced oxidative stress. Bars indicate means ± SD of replicates (n = 3).

Root responses to DAB staining are presented in Figure 6. Lightness (L*, Figure 6a), a* (Figure 6b), and b* (Figure 6c) exhibited noticeable variations, with lower values in Ciech compared to Inow. Hue (Figure 6d) and Chroma (Figure 6e) displayed treatment-related differences, while ΔE and ΔE′ (Figure 6f,g) revealed strong shifts in Soltq roots, particularly between control and 400 mM. The three-dimensional CIELab space (Figure 6h) showed distinctive distributions for each population, with German populations occupying a wider region.

### 2.3. Histochemical Lignin Detection and Colourimetric Analysis

**Figure 7:**
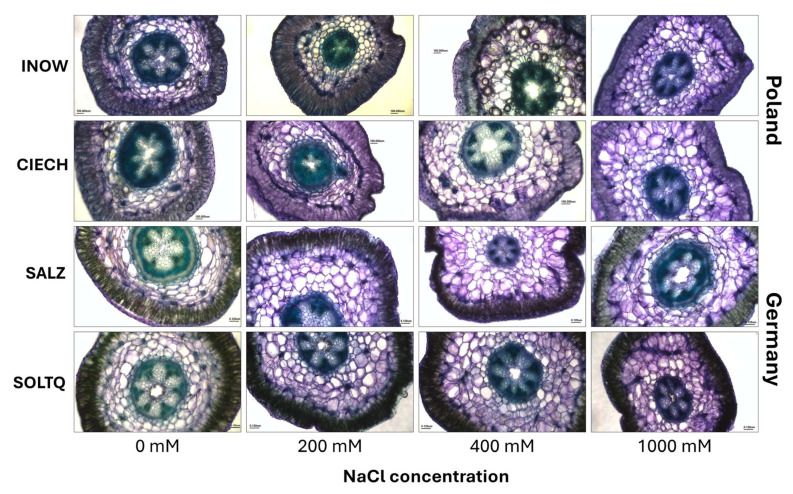
Histochemical detection of lignin in shoots cross-section (scale bar = 200 µm) of *S. europaea* populations (INOW = Inowrocław, CIECH = Ciechocinek, SALZ = Salzgraben, SOLTQ = Soltauquelle) under NaCl stress. (n = 3). Scale bar = 100 m.

Toluidine blue O staining of shoot cross-sections revealed differences in lignin deposition across salinity treatments (Figure 7). Greenish-blue colouration was evident in xylem and interfascicular fibres, with variations in staining intensity among populations.

**Figure 8:**
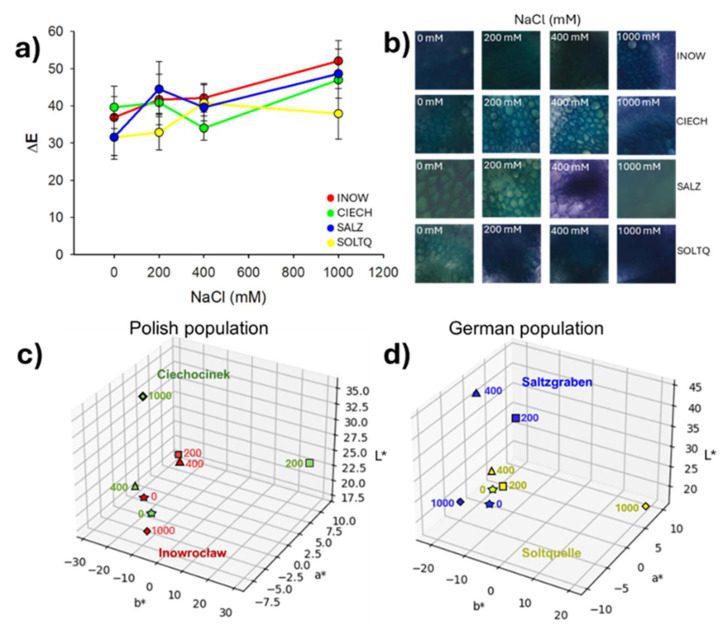
(**a**) Average values of ΔE (total colour difference in lignin determination. Bars indicate means ± SD of replicates (n = 3), (**b**) representative image crops of each stained plant for xylem and phloem zones, (**c**,**d**) 3D plot of the colour changes in L* a* and b* parameters of *S. europaea* populations when subjected to different concentrations of NaCl.

Colourimetric evaluation of lignin staining (Figure 8) showed population- and treatment-specific changes. The overall colour difference (ΔE, Figure 8a) indicated significant shifts at higher salinity in Inow, Ciech and Salz. Figure 8b illustrates representative staining images, where Ciech exhibited more intense greenish-blue colouration. The three-dimensional CIELab plots (Figure 8c,d) displayed broader distributions, mainly in the Polish populations, with Ciech shifting broadly in L* and a*, while Inow formed a more compact cluster, whereas Soltq exhibited wider dispersion across b* values, and Salz formed a more consistent cluster.

Two-way ANOVA (Table S4) confirmed the visual trends: salinity significantly affected the a* and b* axes (*p* = 0.0001 and *p* = 0.0002, respectively), while lightness (L*) showed no significant changes. A significant Population × Salinity interaction was detected only for a* (*p* = 0.0012), indicating that salinity-driven shifts along the red–green axis differed among populations. Collectively, these results demonstrate that lignin deposition, as reflected in CIELab colour profiles, varied across populations and salinity levels, with the most pronounced statistical effects observed for salinity on the a* and b* parameters.

### 2.4. Pectin Distribution and Colourimetric Analysis

**Figure 9:**
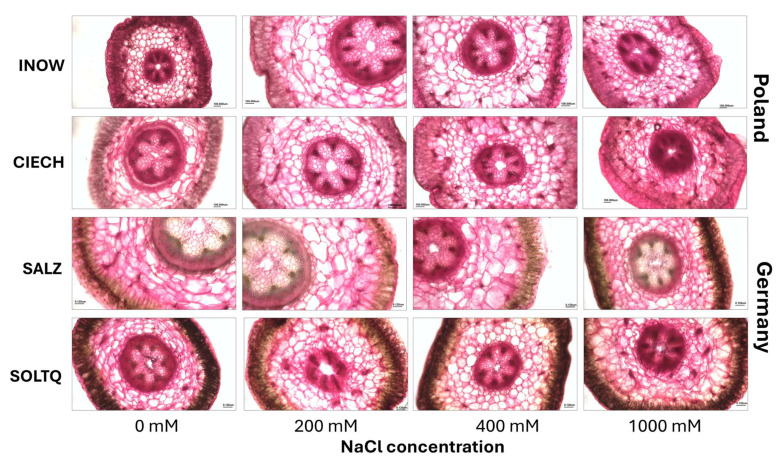
Histochemical detection of pectin in shoots cross-section (scale bar = 200 µm) of *S. europaea* populations (INOW = Inowrocław, CIECH = Ciechocinek, SALZ = Salzgraben, SOLTQ = Soltauquelle) under NaCl stress. (n = 3). Scale bar = 100 μm.

**Figure 10:**
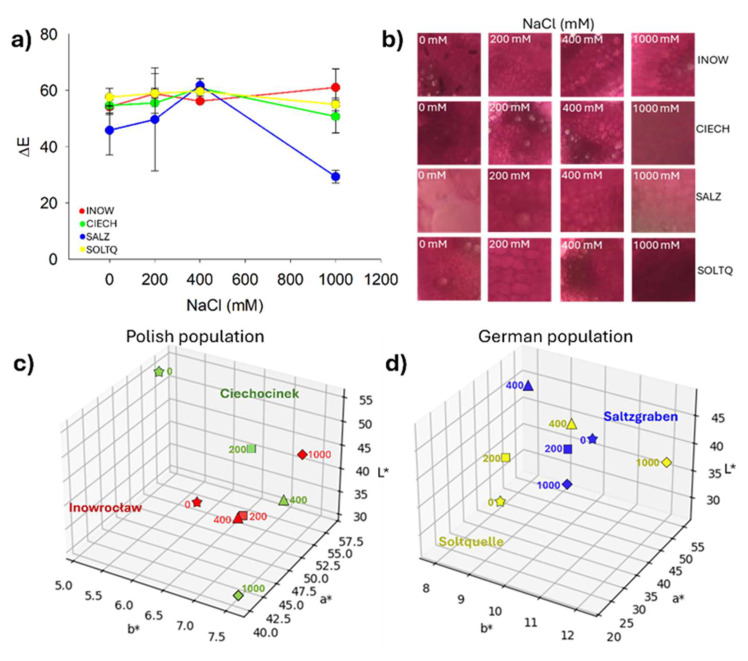
(**a**) Average values of ΔE (total colour difference) for pectin determination are shown. Bars indicate means ± SD of replicates (n = 3). (**b**) Representative image crops of ruthenium-stained plants highlighting xylem and phloem zones, and (**c**,**d**) three-dimensional plots of L*, a*, and b* colour changes in *S. europaea* populations under different NaCl concentrations.

Pectin content was assessed using ruthenium red (Figure 9 and Figure 10). In Figure 9, reddish staining intensity corresponded to pectin levels, with clear differences among treatments and populations.

Quantitative colourimetric changes are presented in Figure 10a, where Inow, Ciech, and Soltq showed steady ΔE values up to 400 mM, followed by sharp increases at 1000 mM. Salz exhibited a strong peak at 400 mM and a decrease at 1000 mM, while Soltq displayed more moderate progressive changes. Representative stained tissues (Figure 10b) illustrated these differences in xylem and phloem. Three-dimensional CIELab distributions (Figure 10c,d) confirmed distinct population trajectories under salinity: Ciech spread broadly across the colour space under stress, while Inow showed a more compact cluster. The German populations, Salz and Soltq, also clustered more compactly, highlighting higher colour stability across treatments.

Two-way ANOVA (Table S4) supported the visual patterns, revealing significant effects of Population on L*, a*, and b* (*p* < 0.01) and of Salinity on all three parameters (*p* ≤ 0.01), with the strongest salinity effect observed for a* (*p* = 0.0001). A significant Population × Salinity interaction was detected for a* (*p* = 0.032), indicating population-specific red–green shifts under salinity, whereas interactions for L* and b* were not significant.

### 2.5. Population Variability Through Colourimetric Parameters

**Figure 11:**
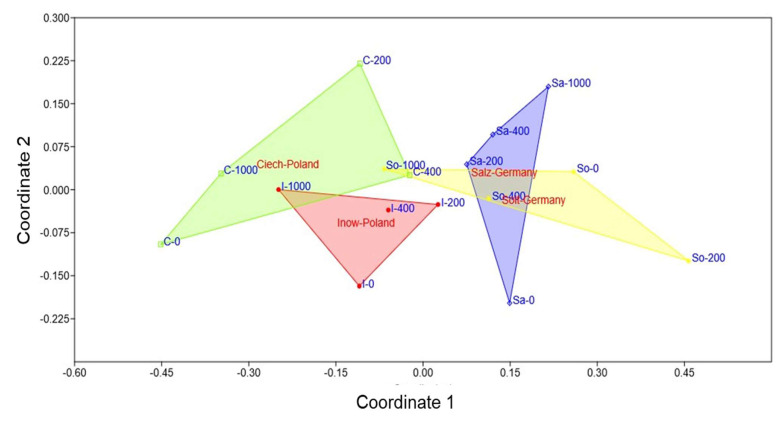
Non-metric multidimensional scaling (NMDS) ordination of four *S. europaea* inland populations (Inowrocław, Ciechocinek, Salzgraben, and Soltauquelle) based on oxidative stress markers (MDA, H_2_O_2_) through CIELab colourimetric parameters under increasing NaCl concentrations (0, 200, 400, and 1000 mM). Polish populations (Ciechocinek—green; Inowrocław—red) cluster on the left, indicating stronger oxidative responses and colour variation. German populations (Salzgraben—blue; Soltauquelle—yellow) cluster on the right, indicating different salinity adaptations, lower oxidative stress colouring and greater stability under salinity.

The NMDS ordination yielded a stress value of 0.143, indicating a good representation of the multivariate data. In two dimensions (Figure 11), this revealed a clear separation among populations. Polish populations clustered on the left, associated with stronger oxidative and histochemical responses, while German populations clustered on the right, indicating more stable profiles. Within the Polish group, Inow extended toward both low and high salinity in a compact cluster, whereas Ciech displayed the wider cluster along Coordinate 1. Among German populations, Salz formed a compact cluster along Coordinate 2, while Soltq showed broader dispersion.

## 3. Discussion

Salinity induces oxidative stress through membrane damage and excessive generation of reactive oxygen species (ROS) [23]. Here, computer vision-based image analysis combined with Schiff’s and DAB staining detected subtle but statistically significant colourimetric changes across salinity treatments and populations, providing a non-destructive and quantitative assessment of oxidative stress severity. Conventional spectrophotometric or chromatographic assays for MDA or H_2_O_2_ are limited by pigment interference, low analyte concentrations, and destructive sampling [24,25]. In contrast, image-based colour analysis offers a robust, cost-efficient alternative that preserves tissue integrity and enables precise, spatially resolved monitoring of stress dynamics [26,27,28], making it a powerful tool for assessing lipid peroxidation and ROS accumulation in halophytes.

The Schiff’s staining patterns (Figure 2; Table 1 and Table 2) revealed population-specific differences in lipid peroxidation, highlighting contrasting oxidative-stress tolerance. Strong staining in Inow and Ciech shoots indicates greater sensitivity to salt-induced oxidative damage, consistent with glycophytes and less salt-adapted halophytes [29,30,31,32] and in line with recent reports of species-specific MDA and H_2_O_2_ dynamics in *S. europaea* under salinity [12,33]. This agrees with the high and variable soil salinity at their maternal sites (ECe ≈ 36.8 dS m^−1^ in Inow and 48.1 dS m^−1^ in Ciech), where anthropogenic enrichment and seasonal fluctuations can trigger ROS overproduction, despite the presence of halophytic traits. In contrast, the reduced staining in Salz shoots reflects a more effective antioxidative defence typical of halophytes from naturally saline habitats [34]. The lower and more stable salinity of the German sites (ECe ≈ 12.7 dS m^−1^ in Salz and 11.0 dS m^−1^ in Soltq) and their long exposure to natural salt springs likely favour constitutive antioxidant systems that limit ROS accumulation. More homogeneous root staining suggests tissue-specific mechanisms linked to reduced ROS generation or efficient ion compartmentalisation [35].

Colourimetric parameters support these patterns: L* and ΔE rose sharply in Polish populations, reflecting rapid pigment bleaching and membrane destabilization [36], whereas the consistently lower L* and higher chroma of Salz indicate pigment preservation via non-enzymatic antioxidants such as carotenoids and flavonoids [37]. Broader CIELab distributions in the Polish populations, compared with the compact clusters of the German populations, highlight how higher and more variable salinity promotes stronger oxidative responses and greater plasticity in Poland, whereas the German populations exhibit constitutive antioxidant strategies under milder, more stable natural salinity conditions.

Root colourimetric profiles (Figure 3a–g) demonstrated more subtle, yet population-specific effects. The broader dispersion in Inow roots compared to the tighter clustering in Ciech suggests different oxidative strategies, with Ciech showing relatively higher stability or negligible lipid peroxidation modifications in the roots compared to the others, as indicated by very low intra-treatment ΔE’ changes (Figure 3g). Among the German populations, Salz roots exhibited greater variability, whereas Soltq remained more stable. These trends align with reports that root-level oxidative responses can reflect habitat-specific soil conditions, with some populations investing in more dynamic ROS regulation while others rely on constitutive stability [35,38].

The DAB staining results in shoots (Figure 4) reveal clear population-specific patterns. Inow and Ciech showed a progressive salinity-dependent increase, with Ciech reaching the highest intensity at 1000 mM. Both German populations stained more strongly at baseline but diverged in trend: Soltq maintained high colouration across all treatments, indicating a constitutive basal H_2_O_2_ pool with minimal change, whereas Salz showed detectable H_2_O_2_ at 0 mM but only a gradual rise with salinity, reflecting tighter redox control.

In the roots, Ciech again displayed a strong inducible increase, while Soltq remained high but stable with only a slight rise at 1000 mM, and Salz retained constitutive H_2_O_2_ with moderate changes across the gradient. Together, these patterns indicate that Soltq sustains high basal H_2_O_2_ in both organs while Salz mounts a more regulated, stepwise response, highlighting the dual nature of H_2_O_2_ in halophytes, where it can act either as a signalling messenger, as an oxidative stressor, or both simultaneously, depending on concentration and environmental conditions [37,39,40].

CIELab parameters associated with DAB staining (Figure 5) paralleled MDA profiles, confirming that H_2_O_2_ accumulation strongly impacts tissue pigmentation. The larger ΔE′ shifts in Inow and Ciech indicate rapid oxidative responses, whereas the more compact 3D CIELab distributions in German populations reflect controlled ROS dynamics. This separation between Polish and German populations illustrates their contrasting oxidative strategies, shaped by local soil salinity and historical exposure [35].

Root H_2_O_2_-associated colourimetric shifts (Figure 6) further highlight these differences. The low resistance in Ciech roots, as indicated by an increase from 0 to 200 mM in L*, and a pronounced decrease in a* values, suggests higher tissue modifications related to oxidative management. Salz roots showed the most pronounced intra-treatment ΔE′ decline at 200 mM, indicating dynamic ROS production before stabilizing at higher levels. The distinct clustering patterns in the 3D colour space reflect adaptive divergence between populations, consistent with the concept of root-specific antioxidant regulation [41].

Toluidine blue O staining (Figure 7) and colourimetric analyses (Figure 8) revealed population-dependent lignin responses. The increased ΔE values in Inow and Salz at higher salinity suggest lignin degradation or redistribution linked to membrane reorganization, a common outcome of ROS-induced peroxidation [42]. In contrast, Soltq exhibited strong colour differences at 1000 mM, consistent with adjustments in vascular lignification, likely associated with nucleic acid content [43]. These contrasting lignin patterns emphasize how cell wall remodelling contributes to salinity adaptation across populations.

Ruthenium red staining (Figure 10 and Figure 11) demonstrated clear differences in pectin responses. The sharp ΔE peak in Salz at 400 mM, followed by a decline at 1000 mM, suggests active remodelling, potentially linked to pectin de-esterification and ion sequestration in the apoplast [44,45]. In contrast, Inow, Ciech, and Soltq showed more progressive changes, but with structural weakening at 1000 mM, suggesting lower remodelling capacity. Soltq exhibited moderate and gradual responses, indicative of a more balanced adjustment strategy.

These trends highlight the role of lignin and pectin dynamics in maintaining cell-wall elasticity and ionic homeostasis during salt stress [18,46]. Importantly, such mechanisms are directly relevant to crop engineering: targeted modification of cell-wall polymers, such as manipulating pectin methylesterification to enhance ion binding or tuning lignin biosynthesis to adjust wall rigidity, has been proposed as a strategy to improve salt resilience in crops, offering practical avenues to transfer insights from *S. europaea* to agricultural species.

The NMDS ordination (Figure 11) produced a two-dimensional solution with a stress value of 0.143, indicating an acceptable representation of the multivariate dissimilarities. It clearly separated Polish and German populations, integrating the oxidative and histochemical markers. The Polish populations clustered with stronger peroxidation, elevated H_2_O_2_, and greater colour shifts, reflecting higher stress sensitivity. By contrast, the German populations, particularly those from Salz, exhibited more stable oxidative and histochemical profiles, indicative of constitutive tolerance. Soltq exhibited broader dispersion, suggesting flexible rather than fixed strategies. This pattern confirms that inland *S. europaea* populations have evolved distinct adaptive strategies, with German populations showing traits consistent with long-term exposure to saline habitats [29,47].

## 4. Materials and Methods

### 4.1. Seed Germination Conditions and Salinity Treatments

*S. europaea* seeds from four inland populations (two in Germany, and two in Poland) were collected and grown under controlled conditions varying salinity levels from 0, 200, 400, and 1000 mM during two months of complete development of *S. europaea*. Then, shoots and roots were carefully selected from the growth species and kept for further analysis.

The *S. europaea* correspond to four spatially separated inland populations which may represent four different ecotypes. In Poland: Inowrocław–Mątwy (Inow) (52°48′ N, 18°15′ E), a salt land with waste from a soda ash and chemical factory (ECe ≈ 36.8 dS m^−1^); Ciechocinek (Ciech) (52°53′ N, 18°47′ E), where saline graduation towers concentrate minerals and salt by evaporation from saline water (ECe ≈ 48.13 dS m^−1^). In Germany: Salzgraben Salzdahlum (Salz) (52°11′56.9″ N, 10°36′05.0″ E), a natural salt spring (ECe ≈ 12.71 dS m^−1^), and Soltauquelle (Soltq) (52°05′24.2″ N, 10°49′18.2″ E), a nature reserve with a natural salt spring that emerges as a pond at the bottom of a valley (ECe ≈ 11.01 dS m^−1^).

*S. europaea* seeds germination was carried out by placing them in Petri dishes (φ = 18 cm) with filter paper moistened with distilled water. Then, a unique seedling was placed in a plastic pot (5.5 cm × 5.3 cm, ~125 cm^3^) for a total of 12 pots per treatment, with a substrate mixture of vermiculite and sand for each salinity treatment. Before planting, each set of 12 pots was placed on individual trays without drainage and fully saturated with 0, 200, 400, and 1000 mM NaCl solutions. To ensure uniform salinity, 600 mL of the prepared solution was added to each tray, allowing for full substrate saturation without standing water, as described by Orzoł et al. [12]. Moreover, to maintain the water level, the trays were watered with distilled water for the first 30 days. Then, to provide nutrients, each tray was watered with 250 mL of Hoagland’s solution (pH = 7) every 1–2 days. The germination and growth of *S. europaea* plants were carried out in a growth chamber under 50–60% relative humidity at 20–25 °C in a 16/8 h day to night ratio and photon flux density of 1000 mmol m^−2^ s^−1^ (Figure 1). After 60 days of plant growth, salinity stress levels, as indicated by MDA and H_2_O_2_, were evaluated in shoots and roots. Analyses were performed in the fleshy middle segment of the primary branch of *S. europaea* plants at different treatments. Sections of around 2.5 ± 0.5 cm at 0, 200, 400, and 1000 mM NaCl were obtained using a bi-shave razor blade as reported previously [48]. For each population and salinity treatment, three biological replicates were collected for subsequent MDA and H_2_O_2_ analyses.

### 4.2. Histochemical Localization of MDA and H_2_O_2_

Schiff’s reagent visualized the histochemical distribution of MDA (a product of lipid peroxidation) according to Pompella and Comporti, [49] method with some modifications. Plant tissues were washed in distilled water and placed in Falcon tubes with 10% Schiff’s reagent for 10 min. The containers with plant tissues were incubated for 1 h at room temperature in the dark. The shoots and roots were washed in distilled water and transferred to Falcon tubes with 0.5% sodium metabisulfite solution in 0.05 M HCl for 2 min, during which a brown colour was observed. Subsequently, the plant tissues were washed with distilled water and then placed in a glycerol ethanol storage solution (1:4, %*v*/*v*) before being photographed.

Moreover, the localization of H_2_O_2_ was performed according to the methods described by Sekulska-Nalewajko et al. and Thordal-Christensen et al. [50,51] standard protocol. Fresh tissue samples were incubated in a 1 mg mL^−1^ solution of 3,3′-diaminobenzidine tetrahydrochloride (3,3′-DAB-HCl), adjusted to pH 3.8, for 8 h and incubated in the growth chamber prior to sampling. The latter samples were placed and stored in a 96% ethanol solution before being photographed. H_2_O_2_ production was visualized as a reddish-brown colouration.

### 4.3. Pectin and Lignin Determination

Fresh plant tissue sections were obtained from the middle portion of the primary branches (fleshy shoot segments) of *S. europaea* plants subjected to 0, 200, 400, and 1000 mM NaCl treatments. Three biological replicates per population and salinity treatment were prepared. Transverse sections (150 µm thick) were cut using a Leica VT1000S vibratome. The most intact and sharply defined slices were selected for further observation. Pectin in cell walls was visualized after incubation of plant tissues for 30 min in the dark in a 0.05% Ruthenium Red solution [21]. After incubation, the samples were transferred to distilled water to remove excess dye for observation. On the other hand, lignin content in cell walls was visualized according to Pradhan Mitra & Loqué [22]. Plant tissues were placed in 0.02% toluidine blue-O for 5 min. After incubation, the samples were washed 5 times in distilled water to remove excess dye.

### 4.4. Image and Colour Analysis

**Figure 12:**
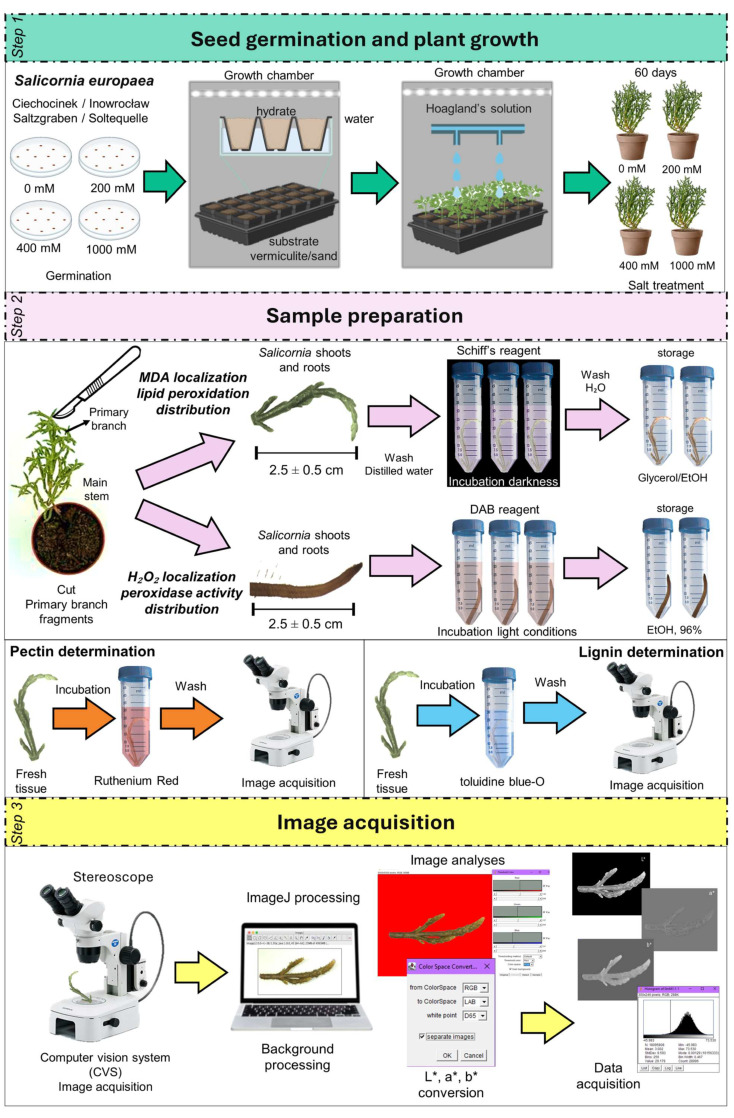
Workflow for image-based analysis of Schiff’s and DAB staining in *S. europaea*. Step 1: Seeds were germinated and plants grown in vermiculite/sand with Hoagland’s solution. Step 2: Primary branches were stained with Schiff’s and DAB reagent and incubated. Step 3: Stained samples were imaged and analyzed via computer vision tools to quantify staining intensity.

For image analysis, a stereomicroscope equipped with a digital camera (Olympus, Hachioji, Japan) was used. The system was connected to a computer for image acquisition, functioning as a Computer Vision System (CVS) (Figure 12). Noise signals were avoided by ensuring the uniform illumination of samples.

RGB images of shoots and roots were acquired and stored in JPEG format with a resolution of 3648 × 2432 pixels (0.1 mm/pixel). For analysis, these images were converted to TIFF format. For each biological replicate and salt treatment, three independent images were captured and analyzed for each of the four populations (Ciech, Inow, Salz, Soltq) to ensure reproducibility. Colour change analysis was performed as described by Cárdenas-Pérez et al. [11,27] reported before. Briefly, the analysis involves capturing, processing, and analyzing the images before and after the treatments. In order to obtain consistent colour data for shoots and roots, all parameters were the same.

To uniformly analyze colour changes in plants exposed to different environmental conditions, the CIELab colour space is considered effective, as it closely aligns with human visual perception [52]. The CIELab space coordinates are represented for: a*: green–red axis; negative values indicate green, and positive values represent orange; b*: blue–yellow axis; negative values indicate blue, and positive values represent yellow; L*: represents luminosity from 0 = black to 100 = white [40,53].

For this reason, images from RGB were transformed into CIELab space using the “Colour space converter” plugin in ImageJ software (v. 1.47, National Institutes of Health, Bethesda, MD, USA) that corresponds to an illuminant of D65. Image colour changes were measured by analyzing 5 images per sample and salt treatment. Before obtaining the L*, a*, and b* values, as explained earlier, the background of the images in TIFF format was removed, and images were transformed to grayscale during the segmentation process by adjusting the threshold. Then, 8-bit images were analyzed using the “Colour space converter” plugin to obtain the individual images corresponding to L*, a*, and b* coordinates. Values were registered by selecting the frequency distribution of pixel intensities from the image histogram. The colour change differences (∆E) calculations were obtained using Equation (1):

(1) $$∆E=\sqrt{{\left({\Delta L}^{*}\right)}^{2}+{\left({∆a}^{*}\right)}^{2}+{\left({∆b}^{*}\right)}^{2}}$$

where ΔL* = L* − L0*; Δa* = a* − a0*; Δb* = b* − b0*, with L0*, a0*, b0* as the colour parameter values of a white reference and L*, a*, b* as the colour parameter values of plants under different salt treatments. These values were compared to those obtained using a colourimeter device (PCE-XXM 30, CM700d, Meschede, Germany) as a reference and were correlated between colourimeter data and image analysis, R^2^ ≥ 0.96, to confirm the accuracy of the colour image analysis.

Additionally, values of chroma (S*) and hue angle (Hue) were calculated using $S^{*}=\sqrt{{(a^{*}}^{2}+{b^{*}}^{2})}$ and $Hue=\arctan\left(\frac{b*}{a*}\right)$, respectively.

### 4.5. Statistical Data Analysis

In order to determine the relationship and differences between all the analyzed species during Schiff reagent lipid peroxidation and DBA for H_2_O_2_ activity, statistical analyses were performed using SigmaPlot software (v. 14.5, SYSTAT Software, Inc., Palo Alto, CA, USA). The analysis was carried out using lipid peroxidation and peroxidase activity as variables, measured through a salinity gradient, in conjunction with colour analysis values obtained from 2D images of the root and shoot. A multivariate analysis of variance (MANOVA) was first applied to the basic CIELab colour parameters (L*, a*, b*), treating Population and Salinity as fixed factors, to evaluate multivariate differences and interactions among treatments (see Supplementary Table S1 for detailed statistics).

Subsequently, two-way ANOVA was performed for each individual colour metric (L*, a*, b*, Hue, S*, ΔE′, and ΔE vs. white) to identify which specific parameters contributed to the multivariate effects and to quantify variability within populations (Table 1 and Table 2) and within salinity levels (Tables S2 and S3 for MDA and H_2_O_2_) and Table S4 for Lignin and Pectin. Analysis was conducted for all results using the Holm–Sidak Test with a significant difference when *p* < 0.05. Moreover, all the 3D colour data plots for CIELab colour changes in Schiff’s lipid peroxidation and DAB-H_2_O_2,_ together with colour changes in histochemical detection of lignin and pectin, were made using Matplotlib codes v3.10.6) a library in Python v.3.11 that allows the creation of graphics by introducing and labelling the data repetitions obtained from image analysis in ImageJ software. Three-dimensional plots were created using the code provided in the Supplementary Information and L*, a*, and b* values obtained from image analysis. Then, the population’s performance along the salinity gradient was compared using Non-metric Multidimensional Scaling (NMDS) with Bray–Curtis dissimilarity based on shoot-based oxidative stress markers (MDA, H_2_O_2_) through CIELab colourimetric parameters. This analysis was performed to compare populations based on highly complex data and to identify the distance between them in the ordination space.

## 5. Conclusions

A comprehensive analysis of *S. europaea* populations under saline conditions revealed that variations in oxidative stress responses are closely linked to distinct biochemical and structural adaptation strategies. Rather than a uniform response, different populations exhibited distinct combinations and magnitudes of lipid peroxidation and H_2_O_2_ production, with cell-wall remodelling referring specifically to the observed variations in pectin and lignin profiles, which differed consistently among populations across the salinity gradient. Although the selected markers are not exclusive to salinity stress, the coordinated patterns and their interaction with site-specific soil salinity (ECe) and population origin provide evidence of population-specific adaptive strategies to salt exposure. In particular, the German populations maintained lower lipid peroxidation and more stable pectin/lignin profiles despite increasing NaCl, consistent with constitutive antioxidant and cell-wall adjustments shaped by their long-term natural salt-spring environments.

Through image-based approaches and histochemical staining, particularly using Schiff’s and DAB reagents, it was possible to visualize and quantify oxidative markers within plant tissues, providing a deeper understanding of cell-wall remodelling and tissue integrity under salt stress. These non-destructive colour-analysis methods proved reproducible and cost-efficient, highlighting their value for future physiological and ecological studies and offering a solid framework that could be further enhanced by artificial intelligence tools for automated detection and pattern recognition in subsequent research.

Notably, the German populations (Salz and Soltq), originating from natural salt springs, showed greater stability and lower oxidative damage than the Polish populations, reflecting constitutive antioxidant defences and long-term ecological adaptation. The observed correlation between lower lipid peroxidation and enhanced stress resilience highlights oxidative-damage mitigation as a key determinant of salt tolerance.

Collectively, these findings clarify the adaptive strategies of the studied *S. europaea* populations and provide practical guidance for the domestication of halophytes and the development of saline agriculture. By combining future AI-driven colour analysis with manipulation of antioxidant capacity and cell-wall polymers, it is possible to accelerate the development and identification of salt-resilient crops and bio-saline remediation systems.

## Figures and Tables

**Table 1:** Two-way ANOVA results from the colour image analysis in *S. europaea* shoot populations under different lipid peroxidation processes through Shiff’s reagent (MDA) and DAB reagent (H_2_O_2_). Populations: INOW, Inowrocław; CIECH, Ciechocinek; SALZ, Salzgraben; SOLTQ, Soltauquelle.

Concentration	Population	L*		a*		b*		Hue		S*		ΔE′		ΔE vs. White Plate	
		Schiff’s-MDA	DAB-H_2_O_2_	Schiff’s-MDA	DAB-H_2_O_2_	Schiff’s-MDA	DAB-H_2_O_2_	Schiff’s-MDA	DAB-H_2_O_2_	Schiff’s-MDA	DAB-H_2_O_2_	Schiff’s-MDA	DAB-H_2_O_2_	Schiff’s-MDA	DAB-H_2_O_2_
0 mM	INOW	45.646 ± 1.58 c	74.02 ± 1.99 a	12.55 ± 0.34 b	14.48 ± 0.34 a	16.19 ± 0.53 b	46.70 ± 3.66 a	35.94 ± 2.42 b	21.14 ± 1.96 c	20.61 ± 0.34 b	46.70 ± 4.13 b	0.00 ± 0.00	0.00 ± 0.00	25.24 ± 0.49 c	44.68 ± 2.68 b
	CIECH	75.53 ± 3.80 b	76.59 ± 2.29 a	13.65 ± 0.27 a	5.08 ± 0.24 c	11.18 ± 0.66 c	48.11 ± 2.33 a	52.20 ± 1.12 a	6.09 ± 0.53 d	17.62 ± 0.48 c	49.71 ± 0.87 a	0.00 ± 0.00	0.00 ± 0.00	20.72 ± 1.35 d	52.23 ± 0.24 a
	SALZ	84.12 ± 3.92 a	45.80 ± 2.60 b	10.12 ± 0.52 c	14.68 ± 1.22 a	15.09 ± 0.52 b	14.85 ± 0.58 b	34.28 ± 1.32 b	47.36 ± 0.20 a	18.37 ± 0.31 c	21.09 ± 0.65 c	0.00 ± 0.00	0.00 ± 0.00	29.03 ± 1.70 b	31.21 ± 0.82 d
	SOLTQ	35.10 ± 2.14 d	27.58 ± 2.11 c	2.51 ± 0.40 d	11.75 ± 0.66 b	27.99 ± 0.86 a	12.19 ± 0.98 b	5.57 ± 0.30 c	41.47 ± 0.86 b	27.40 ± 1.46 a	16.28 ± 1.11 d	0.00 ± 0.00	0.00 ± 0.00	37.85 ± 1.00 a	36.86 ± 2.21 c
200 mM	INOW	83.53 ± 2.56 a	39.41 ± 0.12 b	8.20 ± 0.14 d	23.34 ± 0.27 a	9.56 ± 0.40 c	17.44 ± 0.61 b	220.51 ± 4.44 a	233.09 ± 1.25 a	12.15 ± 0.73 c	29.14 ± 0.42 b	38.54 ± 1.85 a	47.01 ± 3.34 a	26.34 ± 2.53 b	38.87 ± 2.20 a
	CIECH	63.33 ± 1.91 b	52.33 ± 1.80 a	6.69 ± 0.23 c	7.04 ± 0.60 c	15.12 ± 1.19 b	33.23 ± 0.03 a	205.33 ± 3.76 c	189.01 ± 1.49 c	17.57 ± 0.20 b	34.03 ± 0.80 a	15.36 ± 2.55 c	31.30 ± 0.51 b	18.44 ± 0.59 c	34.41 ± 0.95 b
	SALZ	42.01 ± 0.21 c	42.27 ± 1.61 b	2.75 ± 0.50 b	12.62 ± 1.19 b	37.02 ± 5.09 a	17.03 ± 1.81 b	185.02 ± 1.64 d	226.70 ± 8.44 b	36.62 ± 0.85 a	21.96 ± 1.94 c	19.05 ± 1.43 b	27.82 ± 0.95 c	43.64 ± 2.44 a	31.43 ± 9.37 b
	SOLTQ	44.15 ± 1.63 c	35.81 ± 3.11 c	10.54 ± 0.42 a	11.18 ± 0.68 b	15.70 ± 1.06 b	8.00 ± 0.63 c	213.34 ± 2.24 b	230.10 ± 5.95 ab	18.27 ± 1.89 b	13.99 ± 0.40 d	36.87 ± 1.21 a	15.47 ± 0.49 d	26.20 ± 0.22 b	33.86 ± 3.46 b
400 mM	INOW	58.21 ± 0.88 b	54.31 ± 2.68 a	11.20 ± 0.10 a	16.53 ± 1.68 c	15.74 ± 1.44 b	26.76 ± 1.06 a	216.79 ± 3.36 a	210.72 ± 4.14 c	18.81 ± 1.49 b	34.03 ± 3.36 a	22.71 ± 1.64 b	25.29 ± 3.09 b	24.62 ± 1.40 b	36.85 ± 2.83 a
	CIECH	87.06 ± 2.23 a	56.32 ± 2.77 a	4.86 ± 1.19 c	22.93 ± 1.32 a	8.39 ± 0.37 c	25.79 ± 0.70 a	214.65 ± 3.33 a	222.13 ± 0.28 ab	9.40 ± 0.17 d	35.74 ± 1.37 a	10.67 ± 0.38 d	41.02 ± 3.70 a	23.69 ± 2.93 b	36.98 ± 1.49 a
	SALZ	43.32 ± 1.03 d	29.11 ± 0.23 c	9.29 ± 0.14 b	12.14 ± 0.65 d	18.26 ± 0.09 a	11.12 ± 0.62 c	207.85 ± 5.49 b	226.35 ± 1.12 a	21.20 ± 0.69 a	17.22 ± 1.19 c	15.76 ± 0.79 c	4.07 ± 0.99 d	27.99 ± 0.83 a	34.89 ± 1.14 a
	SOLTQ	50.14 ± 1.65 c	43.57 ± 2.58 b	11.66 ± 1.39 a	18.13 ± 1.06 b	14.32 ± 1.30 b	22.55 ± 0.94 b	212.98 ± 4.78 a	217.42 ± 2.18 b	16.77 ± 0.76 c	28.40 ± 0.09 b	25.37 ± 2.28 a	13.42 ± 3.81 c	20.50 ± 1.31 c	31.92 ± 0.24 b
1000 mM	INOW	72.83 ± 2.37 b	44.86 ± 2.11 b	10.69 ± 0.43 a	27.26 ± 0.26 a	11.98 ± 0.54 d	35.43 ± 3.61 b	219.39 ± 3.31 a	224.91 ± 3.83 b	14.93 ± 0.71 b	44.36 ± 1.36 a	29.48 ± 0.44 b	27.63 ± 2.39 a	19.06 ± 0.65 b	44.41 ± 0.16 a
	CIECH	76.59 ± 0.60 a	60.46 ± 0.18 a	10.62 ± 0.29 a	16.51 ± 0.59 b	17.47 ± 2.25 c	41.22 ± 1.87 a	215.67 ± 6.85 ab	201.72 ± 1.74 d	15.89 ± 0.75 b	43.06 ± 0.79 a	14.01 ± 1.56 c	23.79 ± 2.06 b	23.08 ± 0.94 a	44.62 ± 0.86 a
	SALZ	76.56 ± 4.24 a	27.03 ± 1.63 c	11.62 ± 0.47 a	13.98 ± 1.09 c	18.09 ± 1.41 b	11.77 ± 0.38 d	212.87 ± 1.52 b	229.54 ± 2.34 a	22.47 ± 1.07 a	17.76 ± 0.87 c	39.11 ± 2.19 a	3.46 ± 0.99 d	24.12 ± 2.05 a	37.89 ± 0.51 b
	SOLTQ	63.58 ± 2.61 c	45.26 ± 2.69 b	9.70 ± 0.99 b	15.72 ± 0.32 b	20.64 ± 0.27 a	19.97 ± 0.58 c	207.32 ± 3.73 c	220.47 ± 1.89 c	21.79 ± 0.85 a	23.93 ± 1.74 b	27.64 ± 2.10 b	7.74 ± 0.23 c	24.08 ± 0.47 a	27.60 ± 1.94 c

Mean ± standard deviation values with different letters, showing significant differences (*p* < 0.05), replicates (n = 3).

**Table 2:** ANOVA results from the colour image analysis in *S. europaea* roots populations detecting lipid peroxidation and peroxidase activity processes through Shiff’s reagent (MDA) and DAB reagent (H_2_O_2_). Populations: INOW, Inowrocław; CIECH, Ciechocinek; SALZ, Salzgraben; SOLTQ, Soltauquelle.

Concentration	Population	L*		a*		b*		Hue		S*		ΔE′		ΔE vs. White Plate	
		Schiff’s-MDA	DAB-H_2_O_2_	Schiff’s-MDA	DAB-H_2_O_2_	Schiff’s-MDA	DAB-H_2_O_2_	Schiff’s-MDA	DAB-H_2_O_2_	Schiff’s-MDA	DAB-H_2_O_2_	Schiff’s-MDA	DAB-H_2_O_2_	Schiff’s-MDA	DAB-H_2_O_2_
0 mM	INOW	53.88 ± 2.49 b	68.66 ± 0.79 a	−5.35 ± 0.81 a	6.67 ± 0.29 d	22.14 ± 0.17 b	51.92 ± 1.07 a	−15.02 ± 0.62 a	9.15 ± 1.84 d	24.10 ± 2.08 a	52.37 ± 1.34 b	0.00 ± 0.00	0.00 ± 0.00	25.05 ± 1.54 a	54.07 ± 0.40 c
	CIECH	56.55 ± 5.25 b	55.13 ± 1.78 b	−4.45 ± 0.40 a	14.54 ± 1.39 c	25.51 ± 0.36 a	52.15 ± 2.61 a	−9.55 ± 0.28 c	17.30 ± 1.06 c	24.60 ± 2.16 a	53.85 ± 1.63 b	0.00 ± 0.00	0.00 ± 0.00	25.22 ± 2.66 a	54.47 ± 1.61 c
	SALZ	62.13 ± 1.21 a	45.92 ± 1.83 d	−5.39 ± 0.71 a	21.99 ± 0.74 b	20.10 ± 0.22 c	52.25 ± 2.49 a	−15.18 ± 1.91 a	22.21 ± 1.61 b	22.06 ± 2.08 a	56.45 ± 2.44 a	0.00 ± 0.00	0.00 ± 0.00	20.95 ± 0.02 b	58.04 ± 1.89 b
	SOLTQ	66.09 ± 0.34 a	52.19 ± 1.69 c	−4.44 ± 0.37 a	25.09 ± 1.39 a	20.13 ± 0.78 c	53.20 ± 1.48 a	−12.44 ± 0.92 b	25.20 ± 1.63 a	20.61 ± 0.80 b	60.38 ± 3.85 a	0.00 ± 0.00	0.00 ± 0.00	21.30 ± 0.80 b	66.13 ± 2.24 a
200 mM	INOW	60.58 ± 4.11 a	54.92 ± 2.02 a	−3.32 ± 3.51 a	24.01 ± 0.95 a	9.89 ± 0.35 c	16.77 ± 0.91 b	217.18 ± 2.27 a	233.09 ± 1.25 a	12.15 ± 0.73 c	25.81 ± 5.73 b	38.54 ± 1.85 a	47.01 ± 3.34 a	26.34 ± 2.53 b	38.87 ± 2.20 a
	CIECH	55.28 ± 2.55 b	56.39 ± 2.52 a	−4.93 ± 1.07 a	5.37 ± 0.03 c	15.12 ± 1.19 b	32.90 ± 0.54 a	203.33 ± 0.89 c	189.01 ± 1.49 c	17.24 ± 0.73 b	35.03 ± 2.53 a	13.69 ± 3.65 b	31.30 ± 0.51 b	18.44 ± 0.59 c	33.74 ± 2.09 b
	SALZ	64.04 ± 0.61 a	53.18 ± 2.53 a	−4.4 ± 0.34 a	11.68 ± 1.29 b	15.70 ± 1.06 b	8.00 ± 0.63 c	213.34 ± 2.24 b	230.10 ± 5.95 a	18.27 ± 1.89 b	13.99 ± 0.40 d	35.87 ± 1.09 a	15.80 ± 1.05 c	26.20 ± 0.22 b	33.19 ± 2.35 b
	SOLTQ	63.02 ± 2.14 a	47.55 ± 0.31 b	−3.16 ± 1.51 a	12.46 ± 1.43 b	38.35 ± 3.38 a	17.70 ± 2.81 b	183.68 ± 1.35 d	223.37 ± 2.66 b	37.29 ± 1.22 a	21.63 ± 2.21 c	16.72 ± 5.47 b	17.82 ± 0.95 c	43.97 ± 2.46 a	24.76 ± 2.35 c
400 mM	INOW	62.56 ± 2.83 a	64.48 ± 2.22 b	−6.62 ± 0.03 b	10.97 ± 0.32 b	27.38 ± 3.47 a	53.07 ± 0.73 a	162.59 ± 1.44 b	192.32 ± 0.92 b	24.81 ± 3.18 b	52.96 ± 1.69 b	7.41 ± 0.02 a	4.73 ± 1.73 d	24.54 ± 1.13 b	54.23 ± 0.25 b
	CIECH	56.78 ± 2.00 b	66.88 ± 2.99 a	−4.16 ± 0.39 c	2.94 ± 0.80 c	25.89 ± 0.83 a	46.31 ± 1.09 c	170.87 ± 0.77 a	184.44 ± 2.17 c	26.23 ± 0.85 a	46.47 ± 1.11 c	4.81 ± 1.34 b	9.37 ± 0.20 c	27.36 ± 1.96 a	47.69 ± 0.63 c
	SALZ	66.80 ± 2.73 a	35.30 ± 0.73 d	−6.26 ± 0.21 a	26.39 ± 0.83 a	21.90 ± 1.24 b	43.83 ± 1.26 d	162.97 ± 1.61 b	210.20 ± 2.03 a	21.51 ± 1.93 c	52.62 ± 3.78 b	6.48 ± 1.56 a	15.03 ± 0.91 b	22.49 ± 1.75 c	57.57 ± 1.60 a
	SOLTQ	63.11 ± 0.53 a	44.74 ± 0.11 c	−6.83 ± 0.13 a	25.74 ± 1.80 a	19.89 ± 0.68 b	49.79 ± 0.22 b	163.62 ± 2.37 b	207.32 ± 1.74 a	23.49 ± 5.03 b	56.06 ± 0.64 a	3.38 ± 0.17 c	42.20 ± 1.03 a	20.92 ± 0.71 d	58.55 ± 0.64 a
1000 mM	INOW	63.14 ± 1.78 a	63.80 ± 2.30 b	−6.75 ± 0.17 a	5.27 ± 0.58 b	24.03 ± 2.17 b	49.44 ± 3.54 a	164.24 ± 1.19 c	187.08 ± 2.94 c	24.97 ± 2.11 b	49.88 ± 3.89 b	9.05 ± 0.29 a	9.01 ± 2.15 c	25.07 ± 2.14 a	51.76 ± 1.79 b
	CIECH	55.78 ± 0.62 b	67.78 ± 1.43 a	−4.81 ± 0.11 b	6.23 ± 0.60 b	25.74 ± 3.14 a	45.26 ± 2.45 b	173.05 ± 2.62 a	184.70 ± 4.74 c	26.05 ± 3.26 a	45.53 ± 2.69 b	5.40 ± 1.48 b	18.09 ± 0.58 b	25.15 ± 1.18 a	47.08 ± 1.63 c
	SALZ	63.90 ± 2.90 a	44.56 ± 2.21 c	−4.80 ± 0.89 b	23.64 ± 2.83 a	21.27 ± 1.19 c	50.57 ± 0.83 a	167.50 ± 3.25 b	205.94 ± 2.82 b	22.48 ± 1.98 c	54.06 ± 2.79 a	3.46 ± 0.91 b	7.76 ± 1.03 c	21.84 ± 030 b	56.94 ± 2.04 a
	SOLTQ	64.53 ± 2.23 a	31.49 ± 1.22 d	−6.39 ± 0.18 a	22.70 ± 1.04 a	18.72 ± 0.58 d	36.87 ± 1.28 c	162.98 ± 3.04 d	213.16 ± 1.11 a	19.60 ± 0.56 d	46.29 ± 5.34 b	4.03 ± 1.23 b	57.87 ± 0.48 a	20.04 ± 0.60 b	53.28 ± 1.44 a

Mean ± standard deviation values with different letters, showing significant differences (*p* < 0.05), replicates (n = 3).

## Data Availability

Data is contained within the article.

## Supplementary Materials

The following supporting information can be downloaded at: https://www.mdpi.com/article/10.3390/plants14193055/s1, Table S1: Two-way MANOVA results (Wilks’ Lambda, F and *p* values) for colour parameters (L, a, b*) obtained from MDA (Schiff’s reagent) and H_2_O_2_ (DAB) assays in shoots of *S. europaea* populations exposed to different salinity levels; Table S2: ANOVA results from the colour image analysis in the four *S. europaea* shoots populations under MDA-Schiff’s and H2O2-DAB reagents (INOW: Inowrocław, CIECH: Ciechocinek, SALZ: Salzgraben, SOLTQ: Soltquelle). Comparison between saline treatments; Table S3: ANOVA results from the colour image analysis in the *S. europaea* roots populations under under MDA-Schiff’s and H_2_O_2_-DAB reagents (INOW: Inowrocław, CIECH: Ciechocinek, SALZ: Salzgraben, SOLTQ: Soltquelle). Comparison between saline treatments; Table S4: Two-way ANOVA results (F and *p* values) for CIELab colour parameters (L*, a*, b*) derived from pectin (Ruthenium Red staining) and lignin (Toluidine blue-O) assays in shoots of *S. europaea* populations exposed to different salinity levels.

## Abbreviations

The following abbreviations are used in this manuscript:

MDA	Malondialdehyde
H_2_O_2_	Hydrogen peroxide
DAB	3,3′-diaminobenzidine
CVS	Computer vision system
NMDS	Non-metric multi-dimensional scaling
ROS	Reactive oxygen species
INOW	Inowrocław
CIECH	Ciechocinek
SALZ	Salzgraben Salzdahlum
SOLTQ	Soltauquelle
ΔE	Colour difference vs. white plate
ΔE′	Intra-treatment colour difference
