# Supplementary material for: Decoding Salinity Tolerance in Salicornia europaea L.: Image-Based Oxidative Phenotyping and Histochemical Mapping of Pectin and Lignin
Source: Plants (Basel). 2025 Oct 2;14(19):3055. doi: 10.3390/plants14193055 (PMC12526113; doi:10.3390/plants14193055)
Supplement: Supplementary file 1 [file plants-14-03055-s001.zip › plants-3885058-supplementary.pdf]

Supplementary Materials

Susana Dianey Gallegos Cerda [1], Aleksandra Orzło [1], José Jorge Chanona Pérez [2], Josué David Hernández Varela [2], Agnieszka Piernik [1] and Stefany Cárdenas Pérez [1,*]

[1] Department of Geobotany and Landscape Planning, Faculty of Biological and Veterinary Sciences, Nicolaus Copernicus University in Toruń, Lwowska 1, 87-100 Toruń, Poland

[2] Departamento de Ingeniería Bioquímica, Escuela Nacional de Ciencias Biológicas, Instituto Politécnico Nacional, Av. Wilfrido Massieu, Gustavo A. Madero, Ciudad de México 07738, Mexico

* Correspondence: cardenasperez@umk.pl or stefany.cardenasperez@gmail.com

Table S1. Two-way MANOVA results (Wilks' Lambda, *F* and *p* values) for colour parameters (L, a, b*) obtained from MDA (Schiff's reagent) and H_2O_2 (DAB) assays in shoots of *S. europaea* populations exposed to different salinity levels

Effect	MDA Wilks Lambda	*F* value	*p*	H_2O_2 Wilks Lambda	*F* value	*p*
**Population**	0.384	4.96	**<0.001**	0.269	7.37	**<0.001**
**Salinity**	0.905	1.33	0.278	0.673	6.15	**0.0016**
**Population × Salinity**	0.754	1.27	0.266	0.409	4.57	**0.0001**

Table S2. ANOVA results from the colour image analysis in the four *S. europaea* shoots populations under MDA-Schiff's and H_2O_2-DAB reagents (INOW: Inowrocław, CIECH: Ciechocinek, SALZ: Salzgraben, SOLTQ: Soltquelle). Comparison between saline treatments.

Variable	INOW				CIECH			
	0 mM	200 mM	400 mM	1000 mM	0 mM	200 mM	400 mM	1000 mM
L*	45.646 ± 1.58d	83.53 ± 2.56a	58.21 ± 0.88c	72.83 ± 2.37b	75.53 ± 3.80b	63.33 ± 1.91c	87.06 ± 2.23a	76.59 ± 0.60b
	**74.02 ± 1.99a**	**39.41 ± 0.12d**	**54.31 ± 2.68b**	**44.86 ± 2.11c**	**76.59 ± 2.29a**	**52.33 ± 1.80d**	**56.32 ± 2.77c**	**60.46 ± 0.18b**
a*	12.55 ± 0.34a	8.20 ± 0.14d	11.20 ± 0.10b	10.69 ± 0.43c	13.65 ± 0.27a	6.69 ± 0.23c	4.86 ± 1.19d	10.62 ± 0.29b
	**14.48 ± 0.34c**	**23.34 ± 0.27b**	**16.53 ± 1.68c**	**27.26 ± 0.26a**	**5.08 ± 0.24d**	**7.04 ± 0.60c**	**22.93 ± 1.32a**	**16.51 ± 0.59b**
b*	16.19 ± 0.53a	9.56 ± 0.40c	15.74 ± 1.44a	11.98 ± 0.54b	11.18 ± 0.66b	15.12 ± 1.19a	8.39 ± 0.37c	17.47 ± 2.25a
	**46.70 ± 3.66a**	**17.44 ± 0.61d**	**26.76 ± 1.06c**	**35.43 ± 3.61b**	**48.11 ± 2.33a**	**33.23 ± 0.03c**	**25.79 ± 0.70d**	**41.22 ± 1.87b**
Hue	35.94 ± 2.42b	220.51 ± 4.44a	216.79 ± 3.36a	219.39 ± 3.31a	52.20 ± 1.12c	205.33 ± 3.76b	214.65 ± 3.33a	215.67 ± 6.85a
	**21.14 ± 1.96d**	**233.09 ± 1.25b**	**210.72 ± 4.14c**	**224.91 ± 3.83a**	**6.09 ± 0.53d**	**189.01 ± 1.49c**	**222.13 ± 0.28a**	**201.72 ± 1.74b**
S*	20.61 ± 0.34a	12.15 ± 0.73c	18.81 ± 1.49a	14.93 ± 0.71b	17.62 ± 0.48a	17.57 ± 0.20a	9.40 ± 0.17c	15.89 ± 0.75b
	**46.70 ± 4.13a**	**29.14 ± 0.42c**	**34.03 ± 3.36b**	**44.36 ± 1.36a**	**49.71 ± 0.87a**	**34.03 ± 0.80c**	**35.74 ± 1.37c**	**43.06 ± 0.79b**
ΔE'	0.00 ± 0.00	38.54 ± 1.85a	22.71 ± 1.64c	29.48 ± 0.44b	0.00 ± 0.00	15.36 ± 2.55a	10.67 ± 0.38b	14.01 ± 1.56a
	**0.00 ± 0.00**	**47.01 ± 3.34a**	**25.29 ± 3.09b**	**27.63 ± 2.39b**	**0.00 ± 0.00**	**31.30 ± 0.51b**	**41.02 ± 3.70a**	**23.79 ± 2.06c**
ΔE vs white plate	25.24 ± 0.49a	26.34 ± 2.53a	24.62 ± 1.40a	19.06 ± 0.65b	20.72 ± 1.35b	18.44 ± 0.59c	23.69 ± 2.93a	23.08 ± 0.94a
	**44.68 ± 2.68a**	**38.87 ± 2.20b**	**36.85 ± 2.83b**	**44.41 ± 0.16a**	**52.23 ± 0.24a**	**34.41 ± 0.95c**	**36.98 ± 1.49c**	**44.62 ± 0.86b**

Variable	SALZ				SOLTQ			
	0 mM	200 mM	400 mM	1000 mM	0 mM	200 mM	400 mM	1000 mM
L*	84.12 ± 3.92a	42.01 ± 0.21c	43.32 ± 1.03c	76.56 ± 4.24b	35.10 ± 2.14d	44.15 ± 1.63c	50.14 ± 1.65b	63.58 ± 2.61a
	**45.80 ± 2.60a**	**42.27 ± 1.61a**	**29.11 ± 0.23b**	**27.03 ± 1.63b**	**27.58 ± 2.11c**	**35.81 ± 3.11b**	**43.57 ± 2.58a**	**45.26 ± 2.69a**
a*	10.12 ± 0.52b	2.75 ± 0.50c	9.29 ± 0.14b	11.62 ± 0.47a	2.51 ± 0.40c	10.54 ± 0.42a	11.66 ± 1.39a	9.70 ± 0.99b
	**14.68 ± 1.22a**	**12.62 ± 1.19b**	**12.14 ± 0.65b**	**13.98 ± 1.09a**	**11.75 ± 0.66c**	**11.18 ± 0.68c**	**18.13 ± 1.06a**	**15.72 ± 0.32b**
b*	15.09 ± 0.52c	37.02 ± 5.09a	18.26 ± 0.09b	18.09 ± 1.41b	27.99 ± 0.86a	15.70 ± 1.06c	14.32 ± 1.30c	20.64 ± 0.27b
	**14.85 ± 0.58b**	**17.03 ± 1.81a**	**11.12 ± 0.62c**	**11.77 ± 0.38c**	**12.19 ± 0.98c**	**8.00 ± 0.63d**	**22.55 ± 0.94a**	**19.97 ± 0.58b**
Hue	34.28 ± 1.32d	185.02 ± 1.64c	207.85 ± 5.49b	212.87 ± 1.52a	5.57 ± 0.30c	213.34 ± 2.24a	212.98 ± 4.78a	207.32 ± 3.73b
	**47.36 ± 0.20c**	**226.70 ± 8.44b**	**226.35 ± 1.12b**	**229.54 ± 2.34a**	**41.47 ± 0.86c**	**230.10 ± 5.95a**	**217.42 ± 2.18b**	**220.47 ± 1.89b**
S*	18.37 ± 0.31c	36.62 ± 0.85a	21.20 ± 0.69b	22.47 ± 1.07b	27.40 ± 1.46a	18.27 ± 1.89c	16.77 ± 0.76c	21.79 ± 0.85b
	**21.09 ± 0.65a**	**21.96 ± 1.94a**	**17.22 ± 1.19b**	**17.76 ± 0.87b**	**16.28 ± 1.11c**	**13.99 ± 0.40d**	**28.40 ± 0.09a**	**23.93 ± 1.74b**
ΔE'	0.00 ± 0.00	19.05 ± 1.43b	15.76 ± 0.79c	39.11 ± 2.19a	0.00 ± 0.00	36.87 ± 1.21a	25.37 ± 2.28c	27.64 ± 2.10b
	**0.00 ± 0.00**	**27.82 ± 0.95a**	**4.07 ± 0.99b**	**3.46 ± 0.99b**	**0.00 ± 0.00**	**15.47 ± 0.49a**	**13.42 ± 3.81a**	**7.74 ± 0.23b**
ΔE vs white plate	29.03 ± 1.70b	43.64 ± 2.44a	27.99 ± 0.83b	24.12 ± 2.05c	37.85 ± 1.00a	26.20 ± 0.22b	20.50 ± 1.31d	24.08 ± 0.47c
	**31.21 ± 0.82c**	**31.43 ± 9.37c**	**34.89 ± 1.14b**	**37.89 ± 0.51a**	**36.86 ± 2.21a**	**33.86 ± 3.46b**	**31.92 ± 0.24c**	**27.60 ± 1.94d**

Results between variables in regular font refer to the lipid peroxidation process, while those in bold refer to the hydrogen peroxide process. Mean ± standard deviation values with different letters in rows showing significant differences ($p < 0.05$).

Table S3. ANOVA results from the colour image analysis in the *S. europaea* roots populations under under MDA-Schiff's and H_2O_2-DAB reagents (INOW: Inowrocław, CIECH: Ciechocinek, SALZ: Salzgraben, SOLTQ: Soltquelle). Comparison between saline treatments.

Variable	**INOW**				**CIECH**			
	0 mM	200 mM	400 mM	1000 mM	0 mM	200 mM	400 mM	1000 mM
L*	53.88 ± 2.49b	60.58 ± 4.11a	62.56 ± 2.83a	63.14 ± 1.78a	56.55 ± 5.25a	55.28 ± 2.55a	56.78 ± 2.00a	55.78 ± 0.62a
	**68.66 ± 0.79a**	**54.92 ± 2.02c**	**64.48 ± 2.22b**	**63.80 ± 2.30b**	**55.13 ± 1.78b**	**56.39 ± 2.52b**	**66.88 ± 2.99a**	**67.78 ± 1.43a**
a*	-5.35 ± 0.81a	-3.32 ± 3.51a	-6.62 ± 0.03b	-6.75 ± 0.17b	-4.45 ± 0.40a	-4.93 ± 1.07a	-4.16 ± 0.39a	-4.81 ± 0.11a
	**6.67 ± 0.29c**	**23.34 ± 0.27b**	**26.76 ± 1.06b**	**35.43 ± 3.61a**	**21.99 ± 0.74c**	**7.04 ± 0.60d**	**25.79 ± 0.70b**	**41.22 ± 1.87a**
b*	22.14 ± 0.17b	9.89 ± 0.35c	27.38 ± 3.47a	24.03 ± 2.17b	25.51 ± 0.36a	15.12 ± 1.19b	25.89 ± 0.83a	25.74 ± 3.14a
	**46.70 ± 3.66a**	**17.44 ± 0.61d**	**26.76 ± 1.06c**	**35.43 ± 3.61b**	**48.11 ± 2.33a**	**33.23 ± 0.03c**	**25.79 ± 0.70d**	**41.22 ± 1.87b**
Hue	-15.02 ± 0.62c	217.18 ± 2.27a	162.59 ± 1.44b	164.24 ± 1.19b	-9.55 ± 0.28c	203.33 ± 0.89a	170.87 ± 0.77b	173.05 ± 2.62b
	**21.14 ± 1.96d**	**233.09 ± 1.25a**	**210.72 ± 4.14c**	**224.91 ± 3.83b**	**6.09 ± 0.53d**	**189.01 ± 1.49c**	**222.13 ± 0.28a**	**201.72 ± 1.74b**
S*	24.10 ± 2.08a	12.15 ± 0.73b	24.81 ± 3.18a	24.97 ± 2.11a	24.60 ± 2.16a	17.24 ± 0.73b	26.23 ± 0.85a	26.05 ± 3.26a
	**46.70 ± 4.13a**	**29.14 ± 0.42c**	**34.03 ± 3.36b**	**44.36 ± 1.36a**	**49.71 ± 0.87a**	**34.03 ± 0.80c**	**35.74 ± 1.37c**	**43.06 ± 0.79b**
ΔE'	0.00 ± 0.00	38.54 ± 1.85a	7.41 ± 0.02c	9.05 ± 0.29b	0.00 ± 0.00	13.69 ± 3.65a	4.81 ± 1.34b	5.40 ± 1.48b
	**0.00 ± 0.00**	**47.01 ± 3.34a**	**25.29 ± 3.09b**	**27.63 ± 2.39b**	**0.00 ± 0.00**	**31.30 ± 0.51b**	**41.02 ± 3.70a**	**23.79 ± 2.06c**
ΔE vs white plate	25.05 ± 1.54a	26.34 ± 2.53a	24.54 ± 1.13a	25.07 ± 2.14a	25.22 ± 2.66a	18.44 ± 0.59b	27.36 ± 1.96a	25.15 ± 1.18a
	**44.68 ± 2.68a**	**38.87 ± 2.20b**	**36.85 ± 2.83b**	**44.41 ± 0.16a**	**52.23 ± 0.24a**	**34.41 ± 0.95c**	**36.98 ± 1.49c**	**44.62 ± 0.86b**

Variable	**SALZ**				**SOLTQ**			
	0 mM	200 mM	400 mM	1000 mM	0 mM	200 mM	400 mM	1000 mM
L*	62.13 ± 1.21a	64.04 ± 0.61a	66.80 ± 2.73a	63.90 ± 2.90a	66.09 ± 0.34a	63.02 ± 2.14b	63.11 ± 0.53b	64.53 ± 2.23a
	**45.92 ± 1.83b**	**53.18 ± 2.53a**	**35.30 ± 0.73c**	**20.10 ± 0.22d**	**52.19 ± 1.69a**	**47.55 ± 0.31b**	**44.74 ± 0.11b**	**31.49 ± 1.22c**
a*	-5.39 ± 0.71b	-4.4 ± 0.34a	-6.26 ± 0.21b	-4.80 ± 0.89a	-4.44 ± 0.37a	-3.16 ± 1.51a	-6.83 ± 0.13b	-6.39 ± 0.18b
	**44.56 ± 2.21a**	**12.62 ± 1.19b**	**11.12 ± 0.62b**	**11.77 ± 0.38b**	**11.75 ± 0.66c**	**11.18 ± 0.68c**	**22.55 ± 0.94a**	**19.97 ± 0.58b**
b*	15.09 ± 0.52b	15.70 ± 1.06b	21.90 ± 1.24a	21.27 ± 1.19a	20.13 ± 0.78b	38.35 ± 3.38a	19.89 ± 0.68b	18.72 ± 0.58c
	**14.85 ± 0.58b**	**17.03 ± 1.81a**	**11.12 ± 0.62c**	**11.77 ± 0.38c**	**12.19 ± 0.98c**	**8.00 ± 0.63d**	**22.55 ± 0.94a**	**19.97 ± 0.58b**
Hue	-15.18 ± 1.91d	213.34 ± 2.24a	162.97 ± 1.61c	167.50 ± 3.25b	-12.44 ± 0.92c	183.68 ± 1.35a	163.62 ± 2.37b	162.98 ± 3.04b
	**47.36 ± 0.20c**	**226.70 ± 8.44b**	**226.35 ± 1.12b**	**229.54 ± 2.34a**	**41.47 ± 0.86c**	**230.10 ± 5.95a**	**217.42 ± 2.18b**	**220.47 ± 1.89b**
S*	22.06 ± 2.08a	18.27 ± 1.89b	21.51 ± 1.93a	22.48 ± 1.98a	20.61 ± 0.80c	37.29 ± 1.22a	23.49 ± 5.03b	19.60 ± 0.56c
	**21.09 ± 0.65a**	**21.96 ± 1.94a**	**17.22 ± 1.19b**	**17.76 ± 0.87b**	**16.28 ± 1.11c**	**13.99 ± 0.40d**	**28.40 ± 0.09a**	**23.93 ± 1.74b**
ΔE'	0.00 ± 0.00	35.87 ± 1.09a	6.48 ± 1.56b	3.46 ± 0.91c	0.00 ± 0.00	16.72 ± 5.47a	3.38 ± 0.17b	4.03 ± 1.23b
	**0.00 ± 0.00**	**27.82 ± 0.95a**	**4.07 ± 0.99b**	**3.46 ± 0.99b**	**0.00 ± 0.00**	**15.47 ± 0.49a**	**13.42 ± 3.81a**	**7.74 ± 0.23b**
ΔE vs white plate	20.95 ± 0.02c	26.20 ± 0.22a	22.49 ± 1.75b	21.84 ± 0.30b	21.30 ± 0.80b	43.97 ± 2.46a	20.92 ± 0.71b	20.04 ± 0.60b
	**31.21 ± 0.82c**	**31.43 ± 9.37c**	**34.89 ± 1.14b**	**37.89 ± 0.51a**	**36.86 ± 2.21a**	**33.86 ± 3.46a**	**31.92 ± 0.24b**	**27.60 ± 1.94c**

Results between variables in regular font refer to the lipid peroxidation process, while those in bold refer to the hydrogen peroxide process. Mean ± standard deviation values with different letters in rows showing significant differences ($p < 0.05$).

Table S4. Two-way ANOVA results (F and *p* values) for CIELab colour parameters (L*, a*, b*) derived from pectin (Ruthenium Red staining) and lignin (Toluidine blue-O) assays in shoots of *S. europaea* populations exposed to different salinity levels

**Parameter**	**Effect**	***F* value (Lignin)**	***p***	***F* value (Pectin)**	***p***
**L***	Population	2.72	0.061	**4.57**	**0.009**
	Salinity	0.99	0.41	**5.22**	**0.0048**
	Pop × Salinity	1.64	0.147	1.93	0.0826
**a***	Population	0.45	0.722	**4.93**	**0.0063**
	Salinity	**9.5**	**0.00012**	**9.05**	**0.00017**
	Pop × Salinity	**4.2**	**0.00119**	**2.43**	**0.0315**
**b***	Population	1.59	0.211	**18.5**	**3.80E-07**
	Salinity	**8.47**	**0.00028**	**4.21**	**0.0128**
	Pop × Salinity	2.06	0.065	2.05	0.0651

3D plot code of the colour changes in L* a* and b* parameters

```
import matplotlib.pyplot as plt
from mpl_toolkits.mplot3d import Axes3D
plt.savefig('plot.png')

# Data for the first series

x1 = [x1, x2, x3, x4]

y1 = [y1, y2, y3, y4]

z1 = [z1, z2, z3, z4]


# Data for the second series

x2 = [x1, x2, x3, x4]

y2 = [y1, y2, y3, y4]

z2 = [z1, z2, z3, z4]
```

```
# Data for the Third series

x3 = [x1, x2, x3, x4]

y3 = [y1, y2, y3, y4]

z3 = [z1, z2, z3, z4]


# Data for the Fourth series

x4 = [x1, x2, x3, x4]

y4= [y1, y2, y3, y4]

z4= [z1, z2, z3, z4]


# Create the figure
fig = plt.figure()


# Create a 3D object
ax = fig.add_subplot(111, projection='3d')

# Create the first 3D plot (second data series)
ax.scatter(x1, y1, z1, color='r', label='Series 1')


# Create the second 3D plot (second data series)
ax.scatter(x2, y2, z2, color='g', label='Series 2')


# Create the third 3D plot (second data series)
ax.scatter(x3, y3, z3, color='b', label='Series 3')

# Create the third 3D plot (second data series)
ax.scatter(x4, y4, z4, color='y', label='Series 3')


# Create the figure
fig = plt.figure()


# Axis labels
ax.set_xlabel('b*')
ax.set_ylabel('a*')
ax.set_zlabel('L*')


# Title of the plot
ax.set_title('L*, a* and b* parameters')
```

```
# Show the plot
plt.show()
# Enable interactive mode
plt.ion()
```
